# Whole-body staging of female patients with recurrent pelvic malignancies: Ultra-fast ^18^F-FDG PET/MRI compared to ^18^F-FDG PET/CT and CT

**DOI:** 10.1371/journal.pone.0172553

**Published:** 2017-02-22

**Authors:** Julian Kirchner, Lino Morris Sawicki, Saravanabavaan Suntharalingam, Johannes Grueneisen, Verena Ruhlmann, Bahriye Aktas, Cornelius Deuschl, Ken Herrmann, Gerald Antoch, Michael Forsting, Lale Umutlu

**Affiliations:** 1 Department of Diagnostic and Interventional Radiology, University Dusseldorf, Medical Faculty, Dusseldorf, Germany; 2 Department of Diagnostic and Interventional Radiology and Neuroradiology, University Hospital Essen, University of Duisburg-Essen, Essen, Germany; 3 Department of Nuclear Medicine, University Hospital Essen, University of Duisburg-Essen, Essen, Germany; 4 Department of Gynecology and Obstetrics, University Hospital Essen, University of Duisburg-Essen, Essen, Germany; Karl-Franzens-Universitat Graz, AUSTRIA

## Abstract

**Objectives:**

To evaluate the diagnostic feasibility of an ultra-fast ^18^F-FDG PET/MRI protocol, including T2-w and contrast-enhanced T1-w imaging as well as metabolic assessment (PET) in comparison to ^18^F-FDG PET/CT and CT for whole-body staging of female patients with suspected recurrence of pelvic malignancies.

**Methods:**

43 female patients with suspected tumor recurrence were included in this study. Suspicion was based on clinical follow-up and abnormal findings on imaging follow-up. All patients underwent a PET/CT and a subsequent PET/MRI examination. Two readers were asked to evaluate ultra-fast PET/MRI, PET/CT as well as CT datasets of PET/CT separately for suspect lesions regarding lesion count, lesion localization and lesion characterization. Statistical analyses were performed both, on a per-patient and a per-lesion basis.

**Results:**

Tumor relapse was present in 38 of the 43 patients. Based on CT readings 25/38 tumor relapses were correctly identified. PET/CT enabled correct identification of 37/38 patients, PET/MRI correctly identified 36 of the 38 patients with recurrent cancer. On a lesion-based analysis PET/MRI enabled the correct detection of more lesions, comprising a lesion-based sensitivity, specificity, positive predictive value, negative predictive value and diagnostic accuracy of 50%, 58%, 76%, 31%, and 53% for CT, 97%, 83%, 93%, 94%, and 92% for PET/CT and 98%, 83%, 94%, 94%, and 94% for PET/MRI, respectively. Mean scan duration of ultra-fast PET/MRI, PET/CT and whole-body CT amounted to 18.5 ± 1 minutes, 18.2 ± 1 minutes and 3.5 minutes, respectively.

**Conclusion:**

Ultra-fast PET/MRI provides equivalent diagnostic performance and examination time when compared to PET/CT and superior diagnostic performance to CT in restaging female patients suspected to have recurrent pelvic cancer.

## Introduction

Hybrid imaging in terms of positron emission tomography/computed tomography (PET/CT) has been successfully introduced into oncologic staging and restaging of numerous tumor entities over the last years [1–3]. Due to the combination of metabolic information, derived from the PET-component, and morphologic data, based on whole-body CT, ^18^F-FDG PET/CT has been shown superior to conventional cross-sectional imaging modalities for detection of tumor lesions, in particular for (recurrent) pelvic malignancies [4–6]. However, apart from its superior diagnostic capability, ^18^F-FDG PET/CT is affiliated with significantly increased radiation exposure, when compared to conventional imaging [7]. The implementation of integrated positron emission tomography/magnetic resonance imaging (PET/MRI) offers the opportunity to reduce radiation exposure, while preserving high-quality staging of cancer patients. In addition, high soft-tissue contrast of MRI may be beneficial in some pelvic indications. Currently available data demonstrate an equal diagnostic accuracy in staging of oncologic patients [8–10], particularly in recurrent malignancies of the female pelvis [11,12]. Despite initial approaches [13] to shorten the examination time of PET/MRI studies, the markedly prolonged examination time, caused by the acquisition of a variety of MR sequences, still constitutes a major disadvantage of this modality and restricts the implementation into clinical imaging. Hence, careful selection of the needed MRI sequences to a necessary minimum combined with optimizing PET image acquisition protocols as described in previous studies [14] is a promising concept.

Thus, the objective of this study was to evaluate the diagnostic feasibility of an ultra-fast protocol for ^18^F-FDG PET/MRI, including high-resolution morphological (T1w- and T2w-sequences) and metabolic assessment (PET) compared to ^18^F-FDG PET/CT and CT for whole-body staging of female patients with suspected recurrence of pelvic malignancies. Additionally, the radiation exposure in the different modalities was investigated and compared.

## Materials & methods

### Patients

The study was conducted in conformance with the Declaration of Helsinki and approved by the Ethics Commission of the Medical Faculty of the University Duisburg-Essen (study number 11-4822-BO). All patients underwent a clinically indicated whole-body PET/CT and subsequently an additional whole-body PET/MRI after informed written consent was obtained.

Altogether, 81 female patients were enrolled in this trial. All participants were suspect for a recurrence of pelvic malignancy, based on clinical follow-up (pelvic pain, rise in tumor marker in case of ovarian cancer) as well as abnormal findings on imaging follow-up (Ultrasound, CT, MRI). 43 patients (mean age 55 ± 11 years; range 25–79 years) patients met the inclusion criteria of disease recurrence confirmed either by histopathology or corresponding cross-sectional imaging follow-up and were selected for further analysis.

### PET/CT

Whole-body ^18^F-FDG PET / CT was performed on a Biograph mCT 128 scanner (Siemens, Healthcare GmbH, Erlangen, Germany) after intravenous injection of body-weight adapted mean activity of 234 MBq ± 32 MBq (6.32 ± 0.86 mCi) ^18^F-FDG. To ensure blood glucose levels below 150 mg/dl blood samples were obtained. Whole-body full-dose scans (from skull base to mid thigh) were performed in caudo-cranial scan direction 70 s after 100ml iodinated contrast medium was intravenously administered. The CT component of ^18^F-FDG PET/CT (CT_PET/CT_) was acquired using the manufacturer-supplied dose reduction software CareKV and CareDose 4D (Siemens Healthcare GmbH, Erlangen, Germany) with presets of 120 kV, 210 mAs, an increment of 5 mm, a pitch of 1 and a slice thickness of 5 mm.

PET data acquisition was performed in 5–7 bed positions (2 min per bed position, axial field of view (FOV): 21.8 cm, matrix size 256 x 256 and a Gaussian filter of 4mm Full Width at Half Maximum (FWHM)). Iterative reconstruction (3 iterations and 24 subsets) was applied. Attenuation correction was calculated based on obtained CT datasets.

### PET/MRI

Whole-body ^18^F-FDG PET/MRI examinations were performed on an integrated 3 Tesla PET/MRI scanner (Biograph mMR, Siemens Healthcare GmbH, Erlangen, Germany) and obtained with an average delay of 150 ± 47 min after ^18^F-FDG injection. No additional tracer was injected for the subsequent PET/MRI examinations. Corresponding to PET/CT scan volumes covered skull base to mid-thigh.

PET data acquisition was performed in 4–5 bed positions with a median of 4 min per bed position. PET images were reconstructed using the iterative ordered-subset expectation maximization (OSEM) algorithm, 3 iterations and 21 subsets, a Gaussian filter with 4mm full width at half maximum (FWHM) and a 344 × 344 image matrix. For MR-based attenuation correction a two-point (fat, water) coronal 3D-Dixon-VIBE sequence was performed to generate a four-compartment model (background air, lungs, fat, muscle). MRI data were obtained simultaneously using a 16-channel head-and-neck radiofrequency (RF) coil, a 24-channel spine-array RF coil as well as three-to-five 6-channel flex body-coils, depending on the patient size.

To investigate a (hypothetical) ultra-fast PET/MRI protocol the readers were asked to exclusively read the following sequences (out of a standardized longer protocol) (Table 1):

1. A coronal T1-w 3D-Dixon-VIBE sequence prior contrast administration for attenuation correction only.
2. An axial fat-saturated T2-w HASTE (half Fourier acquisition single shot turbo spin echo)
3. An axial T1-w 3D VIBE (fat-suppressed (fs) volume-interpolated breath-hold examination sequence) after intravenous administration of a gadolinium-based contrast medium (Gadovist; Bayer Healthcare, Germany; 0.1mmol/kg bw).

**Table 1:** Magnetic Resonance Imaging Parameters.

	Orientation	Slice thickness (mm)	Repetition time / Echo time (ms)	Flip angle (°)	Field of view (mm)	Phase of View (%)	Matrix size
T1w VIBE Dixon	Coronal	5	3.6/1.23 (1st) 2.46 (2nd)	10	500	65,6	172 x 79
T2w HASTE	Axial	5	1500/117	160	450	81,3	320 × 211
T1w VIBE post contrast	Axial	3,5	4.08/1.51	9	400	75	512 × 230

T1w VIBE = T1-weighted fat-suppressed volume-interpolated breath-hold examination; T2w HASTE = T2-weighted half Fourier acquisition single shot turbo spin echo

### Image analysis

The imaging datasets of CT of ^18^F-FDG PET/CT, ^18^F-FDG PET/CT and ^18^F-FDG PET/MRI were evaluated on a dedicated OsiriX Workstation (Pixmeo SARL, Bernex, Switzerland) and independently analyzed by two experienced radiologists in hybrid imaging interpretation. All datasets were evaluated in random order. Both readers were informed regarding the primary cancer of the patients, yet remained blinded to patient identity, clinical findings as well as results of prior or follow-up imaging concerning potential suspicion for tumor recurrence. CT_PET/CT_, ^18^F-FDG PET/CT and ^18^F-FDG PET/MRI were separately assessed in random order with a minimum of two weeks apart to avoid recognition bias.

As previously described [12] the following features were independently evaluated based on each dataset (CT_PET/CT_, ^18^F-FDG PET/CT, and ^18^F-FDG PET/MRI): (a) lesion count, (b) lesion localization, (c) lesion characterization (1 benign; 2 malignant), and (d) diagnostic confidence (1 not confident, 2 rather confident, 3 very confident). On the morphological datasets lesion density (intensity), shape and contrast enhancement were taken into account. Lymph nodes with a short axis diameter exceeding 10 mm, a spherical configuration, or strong contrast enhancement were regarded as suspicious. For lesion characterization on PET, visually increased focal FDG-uptake in comparison to background tissue was considered indicative of malignancy for both ^18^F-FDG PET/CT and ^18^F-FDG PET/MRI. In all lesions demonstrating focal ^18^F-FDG uptake the maximum standardized uptake value (SUVmax) was measured by placing a manually drawn polygonal volume of interest (VOI) over each lesion on attenuation-corrected PET images. Additionally, the maximum diameter of all suspicious lesions was determined on CT and on T1w MR images. Any discrepancies between the two readers were resolved in a subsequent consensus reading.

### Reference standard

In accordance with current guidelines, histopathological confirmation of suspected tumor recurrence is not necessarily required for malignancies of the female pelvis. Hence, histopathological verification of disease recurrence was only available in 15 out of 43 patients. As described in previous publications [9,15,16] a modified standard of reference was applied to the remaining cases, comprising previous cross-sectional imaging (mean interval 549 ± 610 days; ^18^F-FDG PET/CT: n = 10; CT: n = 6; MRI: n = 1) as well as follow-up cross-sectional imaging (mean interval of 270 ± 241 days; ^18^F-FDG PET/CT: n = 11; CT: n = 13; MRI: n = 1; ^18^F-FDG PET/MRT: n = 1).

### Radiation dose

Effective dose (ED) due to the ^18^F-FDG PET part of the examination (ED^PET^) was calculated by using the whole body ED coefficient for females recommended by Andersson et al. [17] based on the International Commission on Radiation Protection publication 2007: ED^PET^ = AD^FDG^ x (1,53 x 10^−2^); AD^FDG^ is the administered dose in MBq.

ED due to the CT part of the examination (ED^CT^) was estimated according to a method described by Christner et al. [18] in which the dose-length product and a conversion factor are used.

### Statistical analysis

Statistical analysis was performed using IBM SPSS version 22 (IBM Inc, Armonk, NY, USA). Data are presented as mean values ± standard deviation (SD). Sensitivity, specificity, positive predictive value, negative predictive value and diagnostic accuracy for the detection of local tumor recurrences, lymph node and distant metastases were calculated for CT_PET/CT_, PET/CT and PET/MRI, respectively. The proportion of lesions correctly or falsely rated as malignant or benign or missed on FDG PET/MRI, FDG PET/CT, and CT_PET/CT_ was determined using descriptive statistics.

## Results

Out of the 43 enrolled patients 23 (53.5%) were primarily diagnosed with ovarian cancer, 12 (28%) cervical cancer, 4 (9.5%) endometrium cancer, 3 (7%) vulva cancer, and 1 (2%) vaginal cancer.

### Patient based analysis

PET/CT and subsequent PET/MRI were completed successfully in all 43 patients. 38 (88%) patients were shown to suffer from tumor recurrence according to the reference standard. PET/CT correctly identified 37 out of 38 patients with tumor relapse (97%), while PET/MRI missed 2 patients (95% correct identification) and CT missed 13 patients (67% correct identification). All modalities (CT, PET/CT, and PET/MRI) classified one patient false positive, indicating recurrent cancer, without confirmation in subsequently performed histopathology.

### Lesion-based analysis

In accordance with the reference standard, a total of 154 lesions were included in the final analysis. These comprised 113 (73.4%) malignant and 41 (26.6%) benign lesions based on the reference standard (Table 2).

**Table 2:** Distribution of benign and malignant lesions.

	Malignant	Benign	all
**Lymph nodes**	54 (35%)	11 (7.2%)	65 (42.2%)
**Liver lesions**	13 (8.4%)	10 (6.5%)	23 (14.9%)
**Peritoneal lesions**	23 (14.9%)	2 (1.3%)	25 (16.2%)
**Lung**	5 (3.3%)	3 (1.9%)	8 (5.2%)
**Bone**	7 (4.6%)	-	7 (4.6%)
**Local recurrence**	6 (3.9%)	-	6 (3.9%)
**Others**	5 (3.3%)	15 (9.7%)	20 (13%)
**Total**	113 (73.4%)	41 (26.6%)	154 (100%)

Sensitivity, specificity, positive predictive value, negative predictive value and diagnostic accuracy were 98%, 83%, 94%, 94%, and 94% for PET/MRI, respectively. The corresponding results were 97%, 83%, 93%, 94%, and 92% for PET/CT and 50%, 58%, 76%, 31%, and 53% for CT.

Based on PET/MRI readings 2 malignant lesions were falsely rated as benign, comprising a cystic metastasis next to the aorta and spleen, which was incorrectly identified as a seroma as well as an incipient peritoneal carcinosis restricted to the pelvis with low lesion-to-background contrast. Based on PET/CT readings 3 malignant lesions were missed (gluteal muscle metastasis, 2 lymph nodes). In CT 13 lesions were falsely rated as benign, entailing normal-sized lymph nodes without morphologic criteria indicating malignancy. Additionally, 45 lesions were missed, encompassing very small lymph nodes, liver and peritoneal lesions.

### Examination time and estimated effective dose of ionizing radiation

Mean scan duration of whole-body CT, PET/CT, and PET/MRI amounted to 3.5 minutes, 18.2 ± 1 minutes, and 18.5 ± 1 minutes (including the scout scan, Dixon sequence for attenuation correction, contrast media administration and breath-hold commands, as well as shimming for PET/MRI), respectively (Table 3).

**Table 3:** Scan duration of one whole-body CT, PET/CT examination as well as for ultra-fast protocol for whole-body PET/MR imaging.

	Bed positions	Scan duration (min)
CT		3.5
PET/CT	5–6	18.2 ± 1
PET/MRI	4–5	18.5 ± 1

**Fig 1:**
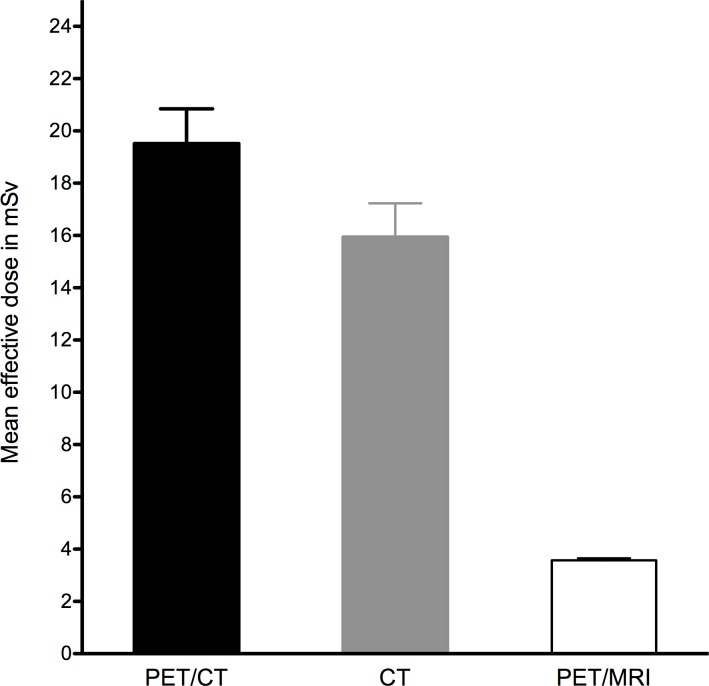
Estimated Effective Dose (ED) of PET/CT, CT, and PET/MRI.

The mean effective dose was calculated as described before and amounted to 15.9 ± 8.5 mSv for whole-body CT and 19.5 ± 8.7 mSv for whole-body PET/CT, consisting of the CT component and effective dose of 3.6 ± 0.5 mSv of the administered ^18^F-FDG (18.5%). Accordingly the mean effective dose for whole-body PET/MRI amounted to 3.6 ± 0.5 mSv (Fig 1).

## Discussion

**Fig 2:**
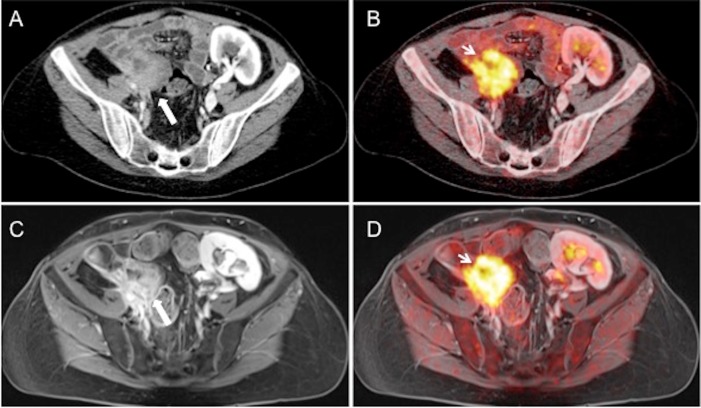
A 46-year-old patient with recurrent cervical cancer. Tumor lesion originating from right ovary with contact to the small bowel is hardly detectable in CT (A). The identical lesion shows a slightly higher lesion contrast in the post-contrast VIBE sequence in MRI (C). In fused PET/CT (B) and PET/MR (D) images the lesion can be equally clearly identified as a metastasis.

Our results demonstrate the feasibility and high diagnostic potential of an ultra-fast PET/MRI protocol in females with suspected recurrent pelvic malignancy. On a lesion-based analysis, ultra-fast PET/MRI and PET/CT showed equivalently high diagnostic performance, while being superior to CT (Fig 2). Furthermore, the implementation of an ultra-fast PET/MRI protocol enables restaging of gynecological cancer patients at comparable short scan duration as in PET/CT, while enabling a significant reduction of the radiation exposure when compared to PET/CT and CT.

PET/CT and PET/MRI have been proven highly valuable in restaging gynecological pelvic cancer due to the combination of metabolic and high-resolution anatomical information [19,20] with previous studies demonstrating the diagnostic equality of PET/MRI compared to PET/CT [12] and diagnostic superiority to MRI alone [11]. Despite excellent diagnostic performance both methods were shown to bear some weak points, namely high radiation exposure in PET/CT and prolonged examination time in PET/MRI. Particularly young patients suffering from tumor entities associated with low mortality rates may be sensitive to the application of repetitive PET/CT examinations with high radiation exposure [21]. The great potential of PET/MRI lies in its multifaceted diagnostic ability. On the one hand multiparametric imaging, requiring dedicated extensive protocols, will be put into focus to provide insights into tumor biology in therapy-naive / therapy-monitored primary tumors [22]. On the other hand, to further enhance the role of PET/MR imaging in clinical routine whole-body imaging, fast efficient protocols are necessary. Initial studies demonstrated the successful implementation of shorter whole-body PET/MRI protocols while preserving the morphologic imaging quality [13,23]. Nevertheless, these studies retained standard PET acquisition times of 4 minutes per bed position, restricting the reduction of the total examination time to a minimum of 26 minutes [13]. Only a few studies investigated the feasibility of truly short PET/MR protocols by utilizing a single sequence, which was originally designed for attenuation-correction, for morphologic correlation [24]. However, this approach degrades the potential of simultaneous PET/MRI to provide the combination of high-resolution morphologic, functional and metabolic information to low-morphologic-metabolic information as in low-dose PET/CT imaging. Hence, the evaluation of ultra-fast PET/MR protocols, entailing the acquisition of high-quality morphologic and metabolic data remains desirable.

Considering recent data demonstrating the questionable added value of diffusion-weighted imaging to PET/MRI as well as a potential reduction of the PET scan time to 2 minutes per bed position (mpb) in PET/MR imaging [14], the aim of this study was to evaluate the diagnostic potential of a hypothetical ultra-fast protocol with preselected sequences from a longer PET/MRI protocol, enabling a significant reduction of the scan time, while preserving high morphological and metabolic data acquisition. Hartung-Knemeyer et al [14] investigated the potential difference in diagnostic quality of PET (derived from PET/MRI), comparing 2 to 8 minutes per bed position PET acquisitions. While the subjective image quality significantly declined from 8 to 2 mpb, neither lesion detectability, nor objective measurements, including signal-to-noise and contrast-to-noise ratio, or SUVmean and SUVmax significantly differed among the acquisition times. Accordingly, we showed the implementation of an ultra-fast PET/MRI protocol, comprising a pre-contrast T1w and T2w sequence as well as a post-contrast (post-PET) T1w sequence within a 2 mpb PET scan time, seems feasible and may lead to a reasonable reduction of the overall scan time to 18.5 ± 1 minutes (including scout scan, shimming, breath hold commands, contrast media application) which would be comparable to PET/CT (comprising 6–7 bed position of 2 mpb).

**Fig 3:**
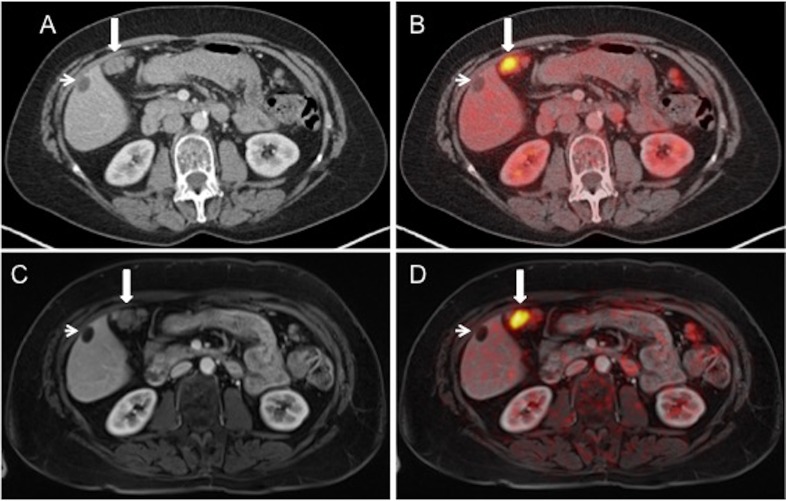
Images of a 58-year-old patient with recurrence of ovarian cancer. CT image (A) shows a peritoneal mass next to the liver with pathological FDG metabolism in fused PET/CT images (B). The same lesion is visible in post-contrast media VIBE sequence in MRI (C) and in PET/MRI (D). No FDG metabolism can be seen in a benign liver cyst in PET/CT or PET/MRI (A-D).

Considering the results of our study, our hypothesis in demonstrating the diagnostic comparability of ultra-fast-PET/MRI to PET/CT and superiority towards CT may be proven valid. While all three imaging modalities falsely rated one patient with perforated sigmoid diverticulitis and reactive FDG-accumulation as indicative for recurrent cancer, both hybrid imaging techniques showed their significant superiority over CT and comparable diagnostic competence among each other. PET/MRI utilizing an ultra-fast protocol demonstrated its diagnostic equivalence to PET/CT, providing slightly inferior sensitivity rates based on patient-based analysis and superior rates based on lesion-based-analysis (Fig 3). Two misinterpreted lesions in PET/MRI leading to misclassifications may be due to the study design, involving subsequent data-acquisition. Due to ethical standards all PET/MRI datasets were obtained after clinically indicated PET/CT examination, resulting in an additional delay of the PET acquisition for PET/MRI. As previously published data demonstrate this delay leads to an increased and altered distribution of FDG accumulation in several parenchymatous organs and background in PET/MRI [25,26]. Accordingly, an increasing FDG accumulation in surrounding non-cancerous tissues and background during the time of prolonged tracer uptake might lead to a decrease of lesion-to-background ratio and false interpretation. Nevertheless, previous publications also reported on increasing FDG accumulation in tumor lesions due to prolonged timing after tracer injection. [27]. However, we did not encounter this in our study. Three missed lesions on PET/CT included a soft tissue metastasis in right gluteal muscle and two inguinal lymph nodes. The soft tissue metastasis was misinterpreted as increased muscle activity because of lacking morphological correlation in CT. Lymph node metastases were not detected with PET/CT because of artifacts due to a hip prosthesis.

Our study is not without some limitations. Investigating a hypothetical ultra-fast-PET/MR protocol, the analyzed sequences were selected from prolonged examination protocols, comprising a median of 4 minute PET acquisition time per bed position. While the investigation of a “true” ultra-fast-protocol would have been desirable, our primary responsibility lies in proper patient care and diagnostics, hence performing a non-evaluated, potentially insufficient ultra-fast-protocol would not have merited our ethical standards. Hence, our study results should be considered as a platform to utilize future studies and evaluate true ultra-fast PET/MR imaging. Secondly, due to our demanding standards regarding long follow-up periods and / or histopathological confirmation for reference standard, the enrolled and analyzed patient cohort is rather small. Hence, our results should be regarded as preliminary and future studies with larger patient cohorts should be performed.

In conclusion, our study results demonstrate the high diagnostic potential of an ultra-fast PET/MR protocol, shortening the examination time to PET/CT acquisition times while preserving high quality MR morphological imaging and equivalent diagnostic performance when compared to PET/CT. This may be of particular interest in case of younger patients, for therapy monitoring as well as long-term aftercare of patients suffering from tumor entities that are associated to low mortality rates, such as early stage cervical cancer or lymphoma. In addition, radiation exposure can be markedly reduced with PET/MRI compared to full-dose PET/CT and CT. With regard to patient comfort and an improved implementation of PET/MRI into routine clinical imaging, ultra-fast PET/MRI may play a crucial role for the diagnostic work-up of cancer patients.

## References

[1] Czernin J, Allen-Auerbach M, Schelbert HR (2007) Improvements in cancer staging with PET/CT: literature-based evidence as of September 2006. J Nucl Med 48 Suppl 1: 78S–88S. 17204723

[2] Beyer T, Townsend DW, Brun T, Kinahan PE, Charron M, Roddy R, et al (2000) A combined PET/CT scanner for clinical oncology. J Nucl Med 41: 1369–1379. 10945530

[3] Antoch G, Vogt FM, Freudenberg LS, Nazaradeh F, Goehde SC, Barkhausen J, et al (2003) Whole-body dual-modality PET/CT and whole-body MRI for tumor staging in oncology. JAMA 290: 3199–3206. 10.1001/jama.290.24.3199 14693872

[4] Kitajima K, Murakami K, Yamasaki E, Domeki Y, Kaji Y, Morita S, et al (2009) Performance of integrated FDG-PET/contrast-enhanced CT in the diagnosis of recurrent uterine cancer: comparison with PET and enhanced CT. Eur J Nucl Med Mol Imaging 36: 362–372. 10.1007/s00259-008-0956-1 18931841

[5] Kitajima K, Murakami K, Yamasaki E, Domeki Y, Kaji Y, Fukasawa I, et al (2008) Performance of integrated FDG-PET/contrast-enhanced CT in the diagnosis of recurrent ovarian cancer: comparison with integrated FDG-PET/non-contrast-enhanced CT and enhanced CT. Eur J Nucl Med Mol Imaging 35: 1439–1448. 10.1007/s00259-008-0776-3 18418592

[6] Gu P, Pan LL, Wu SQ, Sun L, Huang G (2009) CA 125, PET alone, PET-CT, CT and MRI in diagnosing recurrent ovarian carcinoma: a systematic review and meta-analysis. Eur J Radiol 71: 164–174. 10.1016/j.ejrad.2008.02.019 18378417

[7] Brix G, Nosske D, Lechel U (2014) Radiation exposure of patients undergoing whole-body FDG-PET/CT examinations: an update pursuant to the new ICRP recommendations. Nuklearmedizin 53: 217–220. 10.3413/Nukmed-0663-14-04 24919708

[8] Spick C, Herrmann K, Czernin J (2016) 18F-FDG PET/CT and PET/MRI Perform Equally Well in Cancer: Evidence from Studies on More Than 2,300 Patients. J Nucl Med 57: 420–430. 10.2967/jnumed.115.158808 26742709 PMC5003572

[9] Sawicki LM, Grueneisen J, Schaarschmidt BM, Buchbender C, Nagarajah J, Umutlu L, et al (2016) Evaluation of (1)(8)F-FDG PET/MRI, (1)(8)F-FDG PET/CT, MRI, and CT in whole-body staging of recurrent breast cancer. Eur J Radiol 85: 459–465. 10.1016/j.ejrad.2015.12.010 26781152

[10] Heusch P, Nensa F, Schaarschmidt B, Sivanesapillai R, Beiderwellen K, Gomez B, et al (2015) Diagnostic accuracy of whole-body PET/MRI and whole-body PET/CT for TNM staging in oncology. Eur J Nucl Med Mol Imaging 42: 42–48. 10.1007/s00259-014-2885-5 25112399

[11] Grueneisen J, Beiderwellen K, Heusch P, Gratz M, Schulze-Hagen A, Heubner M, et al (2014) Simultaneous positron emission tomography/magnetic resonance imaging for whole-body staging in patients with recurrent gynecological malignancies of the pelvis: a comparison to whole-body magnetic resonance imaging alone. Invest Radiol 49: 808–815. 10.1097/RLI.0000000000000086 25010207

[12] Beiderwellen K, Grueneisen J, Ruhlmann V, Buderath P, Aktas B, Heusch P, et al (2015) [(18)F]FDG PET/MRI vs. PET/CT for whole-body staging in patients with recurrent malignancies of the female pelvis: initial results. Eur J Nucl Med Mol Imaging 42: 56–65. 10.1007/s00259-014-2902-8 25223420

[13] Grueneisen J, Schaarschmidt BM, Heubner M, Suntharalingam S, Milk I, Kinner S, et al (2015) Implementation of FAST-PET/MRI for whole-body staging of female patients with recurrent pelvic malignancies: A comparison to PET/CT. Eur J Radiol 84: 2097–2102. 10.1016/j.ejrad.2015.08.010 26321491

[14] Hartung-Knemeyer V, Beiderwellen KJ, Buchbender C, Kuehl H, Lauenstein TC, Bockisch A, et al (2013) Optimizing positron emission tomography image acquisition protocols in integrated positron emission tomography/magnetic resonance imaging. Invest Radiol 48: 290–294. 10.1097/RLI.0b013e3182823695 23399811

[15] Buchbender C, Hartung-Knemeyer V, Beiderwellen K, Heusch P, Kuhl H, Lauenstein TC, et al (2013) Diffusion-weighted imaging as part of hybrid PET/MRI protocols for whole-body cancer staging: does it benefit lesion detection? Eur J Radiol 82: 877–882. 10.1016/j.ejrad.2013.01.019 23428414

[16] Beiderwellen K, Gomez B, Buchbender C, Hartung V, Poeppel TD, Nensa F, et al (2013) Depiction and characterization of liver lesions in whole body [(1)(8)F]-FDG PET/MRI. Eur J Radiol 82: e669–675. 10.1016/j.ejrad.2013.07.027 24011443

[17] Andersson M, Johansson L, Minarik D, Leide-Svegborn S, Mattsson S (2014) Effective dose to adult patients from 338 radiopharmaceuticals estimated using ICRP biokinetic data, ICRP/ICRU computational reference phantoms and ICRP 2007 tissue weighting factors. EJNMMI Phys 1: 9 10.1186/2197-7364-1-9 26501451 PMC4545621

[18] Christner JA, Kofler JM, McCollough CH (2010) Estimating effective dose for CT using dose-length product compared with using organ doses: consequences of adopting International Commission on Radiological Protection publication 103 or dual-energy scanning. AJR Am J Roentgenol 194: 881–889. 10.2214/AJR.09.3462 20308486

[19] Kim SK, Choi HJ, Park SY, Lee HY, Seo SS, Yoo CW, et al (2009) Additional value of MR/PET fusion compared with PET/CT in the detection of lymph node metastases in cervical cancer patients. Eur J Cancer 45: 2103–2109. 10.1016/j.ejca.2009.04.006 19403303

[20] Caobelli F, Alongi P, Evangelista L, Picchio M, Saladini G, Rensi M, et al (2016) Predictive value of (18)F-FDG PET/CT in restaging patients affected by ovarian carcinoma: a multicentre study. Eur J Nucl Med Mol Imaging 43: 404–413. 10.1007/s00259-015-3184-5 26381775

[21] Torre LA, Bray F, Siegel RL, Ferlay J, Lortet-Tieulent J, Jemal A (2015) Global cancer statistics, 2012. CA Cancer J Clin 65: 87–108. 10.3322/caac.21262 25651787

[22] Pinker K, Andrzejewski P, Baltzer P, Polanec SH, Sturdza A, Georg D, et al (2016) Multiparametric [18F]Fluorodeoxyglucose/ [18F]Fluoromisonidazole Positron Emission Tomography/ Magnetic Resonance Imaging of Locally Advanced Cervical Cancer for the Non-Invasive Detection of Tumor Heterogeneity: A Pilot Study. PLoS One 11: e0155333 10.1371/journal.pone.0155333 27167829 PMC4864307

[23] Grueneisen J, Sawicki LM, Schaarschmidt BM, Suntharalingam S, von der Ropp S, Wetter A, et al (2016) Evaluation of a Fast Protocol for Staging Lymphoma Patients with Integrated PET/MRI. PLoS One 11: e0157880 10.1371/journal.pone.0157880 27327617 PMC4915683

[24] Kohan AA, Kolthammer JA, Vercher-Conejero JL, Rubbert C, Partovi S, Jones R, et al (2013) N staging of lung cancer patients with PET/MRI using a three-segment model attenuation correction algorithm: initial experience. Eur Radiol 23: 3161–3169. 10.1007/s00330-013-2914-y 23765261

[25] Kershah S, Partovi S, Traughber BJ, Muzic RF Jr., Schluchter MD, O'Donnell JK, et al (2013) Comparison of standardized uptake values in normal structures between PET/CT and PET/MRI in an oncology patient population. Mol Imaging Biol 15: 776–785. 10.1007/s11307-013-0629-8 23632951 PMC4822407

[26] Drzezga A, Souvatzoglou M, Eiber M, Beer AJ, Furst S, Martinez-Moller A, et al (2012) First clinical experience with integrated whole-body PET/MR: comparison to PET/CT in patients with oncologic diagnoses. J Nucl Med 53: 845–855. 10.2967/jnumed.111.098608 22534830

[27] Law WP, Maggacis N, Jeavons SJ, Miles KA (2016) Concordance of 18F-FDG PET Uptake in Tumor and Normal Tissues on PET/MRI and PET/CT. Clin Nucl Med. 10.1097/RLU.0000000000001514 28033217

